# Identification of biomarkers from mass spectrometry data using a "common" peak approach

**DOI:** 10.1186/1471-2105-7-358

**Published:** 2006-07-26

**Authors:** Tadayoshi Fushiki, Hironori Fujisawa, Shinto Eguchi

**Affiliations:** 1 Department of Mathematical Analysis and Statistical Inference, Institute of Statistical Mathematics, Tokyo, Japan

## Abstract

**Background:**

Proteomic data obtained from mass spectrometry have attracted great interest for the detection of early-stage cancer. However, as mass spectrometry data are high-dimensional, identification of biomarkers is a key problem.

**Results:**

This paper proposes the use of "common" peaks in data as biomarkers. Analysis is conducted as follows: data preprocessing, identification of biomarkers, and application of AdaBoost to construct a classification function. Informative "common" peaks are selected by AdaBoost. AsymBoost is also examined to balance false negatives and false positives. The effectiveness of the approach is demonstrated using an ovarian cancer dataset.

**Conclusion:**

Continuous covariates and discrete covariates can be used in the present approach. The difference between the result for the continuous covariates and that for the discrete covariates was investigated in detail. In the example considered here, both covariates provide a good prediction, but it seems that they provide different kinds of information. We can obtain more information on the structure of the data by integrating both results.

## Background

Mass spectrometry is being used to generate protein profiles from human serum, and proteomic data obtained from mass spectrometry have attracted great interest for the detection of early-stage cancer (for example, [1-3]). Recent advancements in proteomics come from the development of protein mass spectrometry. Matrix-assisted laser desorption and ionization (MALDI) and surface enhanced laser desorption/ionization (SELDI) mass spectrometry provide high-resolution measurements. Mass spectrometry data are ideally continuous data. Some method is required to deal with high-dimensional but small sample-size data, similar to microarray data. An effective methodology for identifying biomarkers in high-dimensional data is thus an important problem.

**Figure 1:**
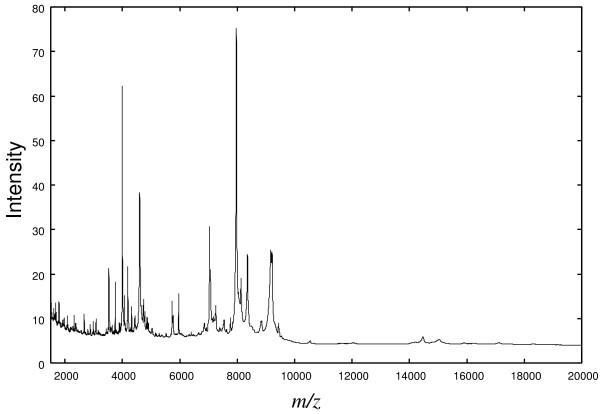
Typical example of proteomic data.

Ovarian Dataset 8-7-02, available from the National Cancer Institute, was analyzed in this paper. This dataset is raw data and consists of 91 controls and 162 ovarian cancer patients. The mass spectrometry data for an individual is illustrated in Fig. 1. The horizontal axis indicates the *m*/*z*-value and the vertical axis the intensity of ion. A characteristic of the data is that a number of peaks can be observed. Each peak represents a singly charged positive ion originating from a protein in the sample. Peaks present in the mass spectrometry data may be usable as biomarkers to judge whether an individual is affected or not.

Some methodologies have been proposed for the identification of biomarkers from ideally continuous mass spectrometry data. One approach is to use peaks in the data to identify biomarkers. Yasui et al.[4] and Tibshirani et al.[5] adopted this approach. Another approach is based on binning of data. Yu et al.[6] and Geurts et al.[7] analyzed mass spectrometry data after binning the data. The methodology presented in this paper adopts the former approach, since peaks in mass spectrometry data are considered to represent biological information. Our idea is that "common" peaks within the sample might contain useful information. This means that a peak seen for only one subject may be noise, whereas a peak exhibited by many subjects might be useful. In this study, an ovarian cancer dataset was analyzed as follows: (1) preprocessing, (2) peak detection, (3) identification of biomarkers, (4) classification. AdaBoost was used to construct a classification function. AsymBoost was also examined for balancing the false negatives and the false positives. The effectiveness of the approach is evaluated using validation data.

The proposed approach is closely related to that of Yasui et al.[4]. In [4], biomarkers were specified through classification by AdaBoost. The present approach differs in that "common" peaks are extracted before classification to specify biomarkers. By specifying biomarkers before classification, the dimension of covariates becomes smaller in classification. We think that it is better if the number of covariates is small in classification. Whereas Yasui et al.[4] used discrete covariates, both continuous covariates and discrete covariates can be used in the present approach, according to the situations. Furthermore, we recommend that the results obtained using discrete covariates should be compared with those obtained using continuous covariates. In the example considered here, more information on the structure of the data can be obtained through the use of both covariates, i.e., different kinds of informative features can be obtained by the use of both covariates.

## Results and discussion

### Dataset

The range of *m*/*z*-value in the dataset is approximately [0, 20000]. However, the frequency of the peaks is too high in the interval [0,1500] and in some cases it is difficult to derive information from peaks in the interval [0,1500]. Therefore, the dataset is analyzed only in the interval [1500, 20000], as in [4].

As is often the case in statistical learning [8], the dataset is divided into two sets; a training dataset that consists of 73 controls and 130 ovarian cancer patients and a test dataset that consists of 18 controls and 32 ovarian cancer patients. The number of the training and test datasets are denoted by *N *and *n*, respectively (*N *= 203 and *n *= 50). The method proposed in this paper is trained using the training dataset, and the performance of the trained scheme is checked using the test dataset.

### Preprocessing

Proteomic data obtained from mass spectrometry are often inaccurate in some senses. For example, the mass/charge axis shift is a big problem in many cases [9,10]. Therefore preprocessing of the data is very important. Preprocessing methods have recently been proposed by Wong et al.[9] and Jeffries [10].

In this paper, preprocessing of the dataset is performed using SpecAlign [9] as follows: (i) subtract baseline, (ii) generate spectrum average, (iii) spectra alignment (peak matching method). It should be noted that it is difficult to align spectra perfectly even if some alignment algorithm is used. In the section of Identification of biomarkers, this problem is reconsidered.

### Peak detection

The peak detection rule of Yasui et al.[4] is adopted here. An *m*/*z *point is regarded as a peak if it takes the maximum value in the *k*-nearest neighborhood. If *k *is small, a point is easily recognized as a peak. An appropriate *k *can be selected by examining some *k*s as done in Yasui et al.[4]. We empirically set *k *= 10. In this study, a slightly small *k *is used, since only the "common" peak is considered as a biomarker.

### Identification of biomarkers

Suppose that some individuals have a peak at a certain *m*/*z*-value, m*. It is then expected that there exists a protein related with the ion corresponding to m*. Therefore, m* may be a biomarker that can be used to judge whether an individual is affected or not. But a peak exhibited by only one subject may just be noise. The peaks "commonly" exhibited by many subjects are thus candidates of biomarkers. However, there remains the problem that the *m*/*z*-values are not perfectly aligned in general, so that the above idea cannot be applied directly. By overcoming this problem of imperfect alignment, the method for identifying such "common peaks" is derived in the following.

**Figure 2:**
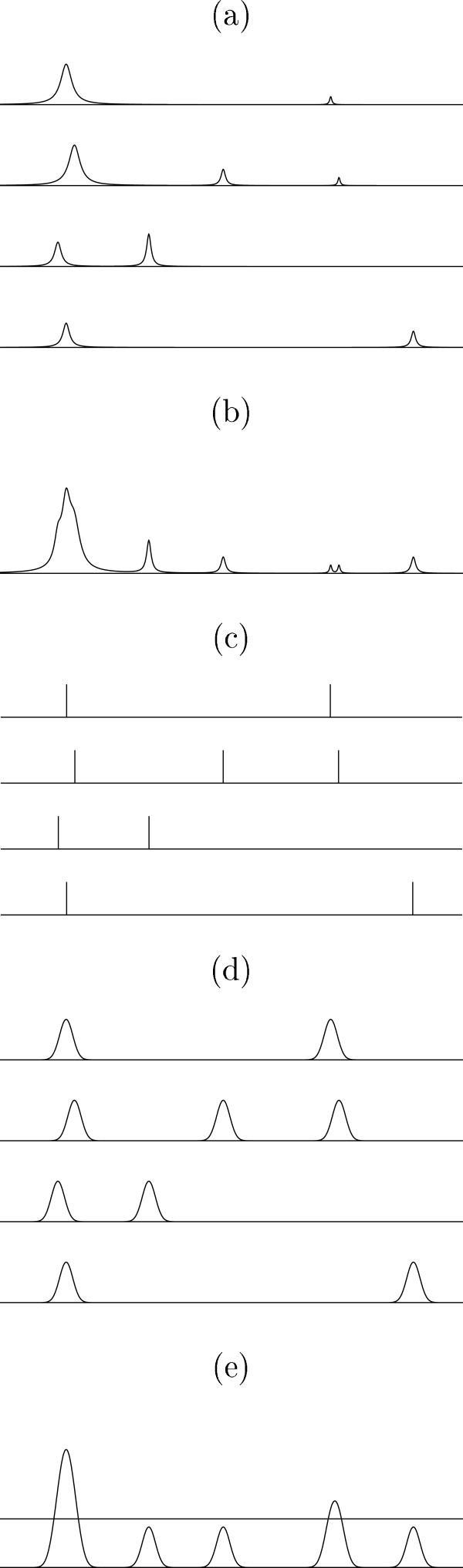
(a) Intensities. (b) Average of intensities. (c) Peaks. (d) Gaussian kernels with centers at peaks. (e) "Average of peaks" with *h*_th _= 0.3.

First, an "average of peaks" is constructed by averaging Gaussian kernels with centers at peaks, as illustrated in Fig. 2. The "average of peaks" is then expressed as

**Figure 3:**
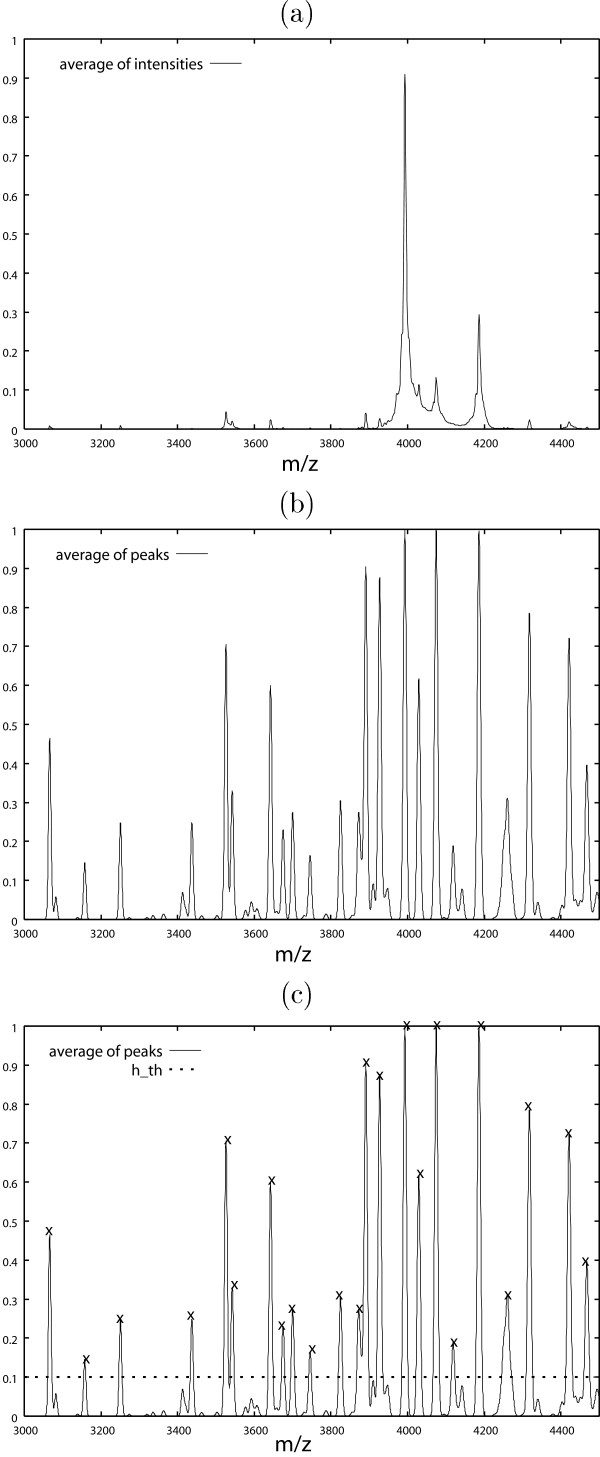
(a) Average of intesities. Intensities are averaged after normalization to [0, 1]. (b) "Average of peaks." (c) Selected biomarkers with *h*_th _= 0.1. In (c), crosses denote biomarkers.

**Figure 4:**
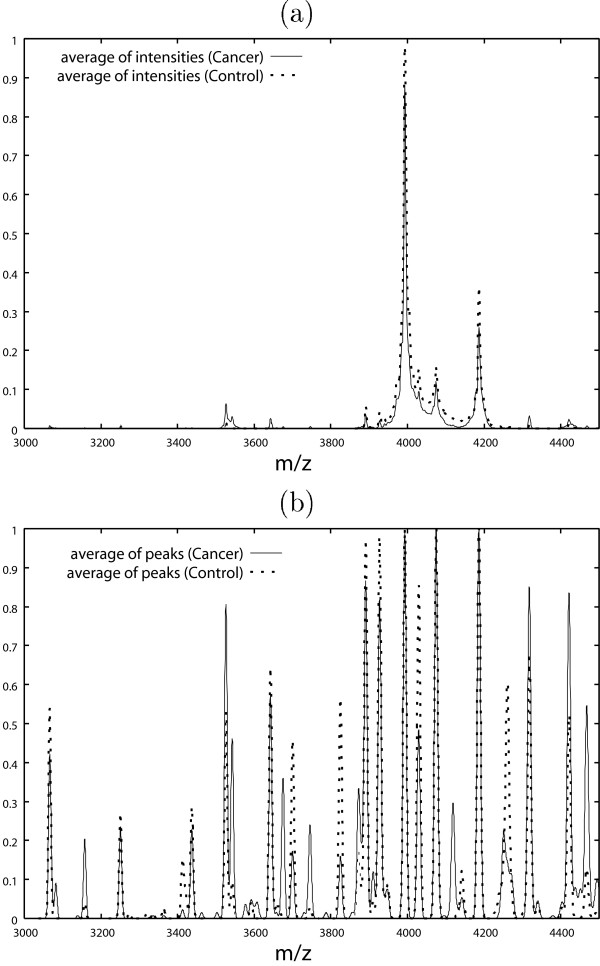
(a) Average of intensities for control and ovarian cancer patient datasets. (b) "Average of peaks" for control and ovarian cancer patient datasets.

A(x)=1N∑i=1N∑jexp⁡[−(x−pi,j)2{σ(pi,j)}2],     (1)
 MathType@MTEF@5@5@+=feaafiart1ev1aaatCvAUfKttLearuWrP9MDH5MBPbIqV92AaeXatLxBI9gBaebbnrfifHhDYfgasaacH8akY=wiFfYdH8Gipec8Eeeu0xXdbba9frFj0=OqFfea0dXdd9vqai=hGuQ8kuc9pgc9s8qqaq=dirpe0xb9q8qiLsFr0=vr0=vr0dc8meaabaqaciaacaGaaeqabaqabeGadaaakeaacqWGbbqqcqGGOaakcqWG4baEcqGGPaqkcqGH9aqpdaWcaaqaaiabigdaXaqaaiabd6eaobaadaaeWbqaamaaqafabaGagiyzauMaeiiEaGNaeiiCaahaleaacqWGQbGAaeqaniabggHiLdaaleaacqWGPbqAcqGH9aqpcqaIXaqmaeaacqWGobGta0GaeyyeIuoakmaadmGabaGaeyOeI0YaaSaaaeaacqGGOaakcqWG4baEcqGHsislcqWGWbaCdaWgaaWcbaGaemyAaKMaeiilaWIaemOAaOgabeaakiabcMcaPmaaCaaaleqabaGaeGOmaidaaaGcbaGaei4EaShcciGae83WdmNaeiikaGIaemiCaa3aaSbaaSqaaiabdMgaPjabcYcaSiabdQgaQbqabaGccqGGPaqkcqGG9bqFdaahaaWcbeqaaiabikdaYaaaaaaakiaawUfacaGLDbaacqGGSaalcaWLjaGaaCzcamaabmGabaGaeGymaedacaGLOaGaayzkaaaaaa@6167@

where *p*_*i*,*j *_is the *m*/*z*-value of the *i*-th observation and the *j*-th peak and *σ *(*p*_*i*,*j*_) shows the "width" of the peak. In this study, *σ *(*p*_*i*,*j*_) = 0.001 × *p*_*i*,*j*_. In general, a very small *σ *is not desirable because the same peaks could not be "added" properly, but a very large *σ *is not also desirable because a peak affects other peaks. The value of *σ *could be decided based on the accuracy of the mass/charge axis. In this study, 2*σ *(*p*_*i*,*j*_)/*p*_*i*,*j *_= 0.002. This corresponds to about ± 0.2% error of the mass/charge value of each peak point. In this dataset, a small change of *σ *did not affect the result. Secondly, a biomarker is identified by finding the peak greater than *h*_th _in the "average of peaks," where *h*_th _is a parameter controlling how "common" peaks can be regarded as biomarkers (Fig. 2 (e)). With *h*_th _= 0.1, 146 biomarkers were obtained by the procedure described in the previous section (Fig. 3 (c)). The features of the approach are as follows:

• In general, peaks cannot be aligned perfectly even if some alignment algorithm is applied, as stated in the section of Preprocessing. In the "average of peaks," even if peaks are not aligned perfectly, they can be added because they have "width" *σ *(*p*_*i*,*j*_) (Figs. 2 (d) and 2 (e)).

• Another possible approach is to use the average of intensities (Fig. 2 (b)). However, we think that the "average of peaks" is more effective in mass spectrometry data (Fig. 2 (e)). "Common" peaks with small intensities can be found easily in Fig. 3 (b), whereas it is difficult to find such "small common" peaks in Fig. 3 (a). Furthermore, the difference between controls and ovarian cancer patients can be seen more clearly in the "average of peaks" than in the average of intensities (Fig. 4).

There are many ways to reduce the number of biomarkers determined by the above procedure. One way is to select biomarkers that are effective in classification. In this study, however, we simply used the biomarkers obtained by the above procedure.

The covariates are extracted from the data as discrete variables and/or continuous variables. Let *m*_1_, *m*_2_, ⋯ be the *m*/*z*-values of the biomarkers. The discrete covariate is obtained by searching for a peak within a window of the biomarker, i.e.,

xj={1,there exists a peak within a window [(1−ρ)mj,(1+ρ)mj],0,otherwise.
 MathType@MTEF@5@5@+=feaafiart1ev1aaatCvAUfKttLearuWrP9MDH5MBPbIqV92AaeXatLxBI9gBaebbnrfifHhDYfgasaacH8akY=wiFfYdH8Gipec8Eeeu0xXdbba9frFj0=OqFfea0dXdd9vqai=hGuQ8kuc9pgc9s8qqaq=dirpe0xb9q8qiLsFr0=vr0=vr0dc8meaabaqaciaacaGaaeqabaqabeGadaaakeaacqWG4baEdaWgaaWcbaGaemOAaOgabeaakiabg2da9maaceqabaqbaeaabiGaaaqaaiabigdaXiabcYcaSaqaaiabbsha0jabbIgaOjabbwgaLjabbkhaYjabbwgaLjabbccaGiabbwgaLjabbIha4jabbMgaPjabbohaZjabbsha0jabbohaZjabbccaGiabbggaHjabbccaGiabbchaWjabbwgaLjabbggaHjabbUgaRjabbccaGiabbEha3jabbMgaPjabbsha0jabbIgaOjabbMgaPjabb6gaUjabbccaGiabbggaHjabbccaGiabbEha3jabbMgaPjabb6gaUjabbsgaKjabb+gaVjabbEha3jabbccaGiabcUfaBjabcIcaOiabigdaXiabgkHiTGGaciab=f8aYjabcMcaPiabd2gaTnaaBaaaleaacqWGQbGAaeqaaOGaeiilaWIaeiikaGIaeGymaeJaey4kaSIae8xWdiNaeiykaKIaemyBa02aaSbaaSqaaiabdQgaQbqabaGccqGGDbqxcqGGSaalaeaacqaIWaamcqGGSaalaeaacqqGVbWBcqqG0baDcqqGObaAcqqGLbqzcqqGYbGCcqqG3bWDcqqGPbqAcqqGZbWCcqqGLbqzcqGGUaGlaaaacaGL7baaaaa@84E6@

The continuous covariate is the maximum value of the intensity within the window.

*x*_*j *_= the maximum value of the intensity within [(1 - *ρ*)*m*_*j*_, (1 + *ρ*)*m*_*j*_].

In this study, *ρ *= 0.002.

### AdaBoost

The present objective is to find the important features of a peak pattern associated with a disease on the basis of peak identification on proteomic data. We introduce AdaBoost for the extraction of informative patterns in the feature space based on examples consisting of *N *pairs of the feature vector and the label. In this context, the feature vector is obtained from peak intensities over the detected *m*/*z*-values for a subject, and the label expresses the disease status of the subject. For pattern classification, one of two cases are employed, that is, in which the feature vector is composed of discrete or continuous values, as discussed in the preceding section.

Ensemble learning has been studied in machine learning. AdaBoost [11] is one of the most efficient learning methods in ensemble learning. As explained below, AdaBoost provides a classification function by a linear combination of weak learners. The AdaBoost algorithm can be regarded as a sequential minimization algorithm for the exponential loss function.

Let *x *be a feature vector and *y *a binary label with values +1 and -1. A classification function *f*(*x*) is then used to predict the label *y*. If *f*(*x*) is positive (or negative), then the label is predicted as +1 (or -1). Suppose that a class of classification functions ℱ
 MathType@MTEF@5@5@+=feaafiart1ev1aaatCvAUfKttLearuWrP9MDH5MBPbIqV92AaeXatLxBI9gBamrtHrhAL1wy0L2yHvtyaeHbnfgDOvwBHrxAJfwnaebbnrfifHhDYfgasaacH8akY=wiFfYdH8Gipec8Eeeu0xXdbba9frFj0=OqFfea0dXdd9vqai=hGuQ8kuc9pgc9s8qqaq=dirpe0xb9q8qiLsFr0=vr0=vr0dc8meaabaqaciaacaGaaeqabaWaaeGaeaaakeaaimaacqWFXeIraaa@3787@ = {*f*} is provided. In AdaBoost, a classification function *f *in ℱ
 MathType@MTEF@5@5@+=feaafiart1ev1aaatCvAUfKttLearuWrP9MDH5MBPbIqV92AaeXatLxBI9gBamrtHrhAL1wy0L2yHvtyaeHbnfgDOvwBHrxAJfwnaebbnrfifHhDYfgasaacH8akY=wiFfYdH8Gipec8Eeeu0xXdbba9frFj0=OqFfea0dXdd9vqai=hGuQ8kuc9pgc9s8qqaq=dirpe0xb9q8qiLsFr0=vr0=vr0dc8meaabaqaciaacaGaaeqabaWaaeGaeaaakeaaimaacqWFXeIraaa@3787@ is called a weak learner. A new classification function *F *is constructed by taking a linear combination of classification functions in ℱ
 MathType@MTEF@5@5@+=feaafiart1ev1aaatCvAUfKttLearuWrP9MDH5MBPbIqV92AaeXatLxBI9gBamrtHrhAL1wy0L2yHvtyaeHbnfgDOvwBHrxAJfwnaebbnrfifHhDYfgasaacH8akY=wiFfYdH8Gipec8Eeeu0xXdbba9frFj0=OqFfea0dXdd9vqai=hGuQ8kuc9pgc9s8qqaq=dirpe0xb9q8qiLsFr0=vr0=vr0dc8meaabaqaciaacaGaaeqabaWaaeGaeaaakeaaimaacqWFXeIraaa@3787@, i.e.,

F(x;β)=∑t=1Tβtft(x),
 MathType@MTEF@5@5@+=feaafiart1ev1aaatCvAUfKttLearuWrP9MDH5MBPbIqV92AaeXatLxBI9gBaebbnrfifHhDYfgasaacH8akY=wiFfYdH8Gipec8Eeeu0xXdbba9frFj0=OqFfea0dXdd9vqai=hGuQ8kuc9pgc9s8qqaq=dirpe0xb9q8qiLsFr0=vr0=vr0dc8meaabaqaciaacaGaaeqabaqabeGadaaakeaacqWGgbGrcqGGOaakcqWG4baEcqGG7aWoiiGacqWFYoGycqGGPaqkcqGH9aqpdaaeWbqaaiab=j7aInaaBaaaleaacqWG0baDaeqaaOGaemOzay2aaSbaaSqaaiabdsha0bqabaGccqGGOaakcqWG4baEcqGGPaqkaSqaaiabdsha0jabg2da9iabigdaXaqaaiabdsfaubqdcqGHris5aOGaeiilaWcaaa@45C6@

where *β *= (*β*_1_, ⋯, *β*_*T*_) is a weight vector and *f*_*t *_∈ ℱ
 MathType@MTEF@5@5@+=feaafiart1ev1aaatCvAUfKttLearuWrP9MDH5MBPbIqV92AaeXatLxBI9gBamrtHrhAL1wy0L2yHvtyaeHbnfgDOvwBHrxAJfwnaebbnrfifHhDYfgasaacH8akY=wiFfYdH8Gipec8Eeeu0xXdbba9frFj0=OqFfea0dXdd9vqai=hGuQ8kuc9pgc9s8qqaq=dirpe0xb9q8qiLsFr0=vr0=vr0dc8meaabaqaciaacaGaaeqabaWaaeGaeaaakeaaimaacqWFXeIraaa@3787@ for *t *= 1, ⋯, *T*. The sign of *F*(*x*; *β*) provides a label prediction of *y*. This is a rule of majority vote by *T *classification functions *f*_1_(*x*), ⋯, *f*_*T*_(*x*) with weights *β*_1_, ⋯, *β*_*T*_. Consider a problem in which weights *β*_1_, ⋯, *β*_*T *_and classification functions *f*_1_(*x*), ⋯,*f*_*T*_(*x*) are optimally combined based on *N *given examples of (*x*_1_, *y*_1_), ⋯, (*x*_*N*_, *y*_*N*_). AdaBoost aims to solve the problem by minimizing the exponential loss defined by

∑i=1Nexp⁡[−yi{β1f1(xi)+⋯+βTfT(xi)}].     (2)
 MathType@MTEF@5@5@+=feaafiart1ev1aaatCvAUfKttLearuWrP9MDH5MBPbIqV92AaeXatLxBI9gBaebbnrfifHhDYfgasaacH8akY=wiFfYdH8Gipec8Eeeu0xXdbba9frFj0=OqFfea0dXdd9vqai=hGuQ8kuc9pgc9s8qqaq=dirpe0xb9q8qiLsFr0=vr0=vr0dc8meaabaqaciaacaGaaeqabaqabeGadaaakeaadaaeWbqaaiGbcwgaLjabcIha4jabcchaWjabcUfaBjabgkHiTiabdMha5naaBaaaleaacqWGPbqAaeqaaOGaei4EaShcciGae8NSdi2aaSbaaSqaaiabigdaXaqabaGccqWGMbGzdaWgaaWcbaGaeGymaedabeaakiabcIcaOiabdIha4naaBaaaleaacqWGPbqAaeqaaOGaeiykaKIaey4kaSIaeS47IWKaey4kaSIae8NSdi2aaSbaaSqaaiabdsfaubqabaaabaGaemyAaKMaeyypa0JaeGymaedabaGaemOta4eaniabggHiLdGccqWGMbGzdaWgaaWcbaGaemivaqfabeaakiabcIcaOiabdIha4naaBaaaleaacqWGPbqAaeqaaOGaeiykaKIaeiyFa0Naeiyxa0LaeiOla4IaaCzcaiaaxMaadaqadiqaaiabikdaYaGaayjkaiaawMcaaaaa@5DDD@

AdaBoost does not jointly provide the optimal solution, but offers a sophisticated learning algorithm with sequential structure involving two stages of optimization in which the best weak learner *f*_*t*_(*x*) is selected in the first stage and the best scalar weight *β*_*t *_is determined in the second stage at the *t*-step.

In this study, decision stumps were used as weak learners. A decision stump is a naive classification function in which, for a subject with a feature vector *x *of peak intensities, the label is predicted by observing whether a certain peak intensity is larger than a predetermined value or not. Accordingly, the set of weak learners is relatively large, but all of the weak learners are literally weak, since they respond only to a peak pattern. The set of the decision stumps is denoted by

{*d*_*j*_(*x*) = sign(*x*_*j *_- *b*) | *j *= 1, ⋯ , *J*, -∞ <*b *< ∞ }.

AdaBoost efficiently integrates the set of weak learners by sequential minimization of the exponential loss. As a result, the learning process of AdaBoost can be traced, and the final classification function can be reexpressed as the sum of the peak pattern functions *F*_*j*_(*x*)'s, where

Fj(x)=∑{t|ft=dj}βt sign (xj−bt),
 MathType@MTEF@5@5@+=feaafiart1ev1aaatCvAUfKttLearuWrP9MDH5MBPbIqV92AaeXatLxBI9gBaebbnrfifHhDYfgasaacH8akY=wiFfYdH8Gipec8Eeeu0xXdbba9frFj0=OqFfea0dXdd9vqai=hGuQ8kuc9pgc9s8qqaq=dirpe0xb9q8qiLsFr0=vr0=vr0dc8meaabaqaciaacaGaaeqabaqabeGadaaakeaacqWGgbGrdaWgaaWcbaGaemOAaOgabeaakiabcIcaOiabdIha4jabcMcaPiabg2da9maaqafabaacciGae8NSdi2aaSbaaSqaaiabdsha0bqabaaabaGaei4EaSNaemiDaqNaeiiFaWNaemOzay2aaSbaaWqaaiabdsha0bqabaWccqGH9aqpcqWGKbazdaWgaaadbaGaemOAaOgabeaaliabc2ha9bqab0GaeyyeIuoakiabbccaGiabbohaZjabbMgaPjabbEgaNjabb6gaUjabbccaGiabcIcaOiabdIha4naaBaaaleaacqWGQbGAaeqaaOGaeyOeI0IaemOyai2aaSbaaSqaaiabdsha0bqabaGccqGGPaqkcqGGSaalaaa@5652@

in which the sum of coefficients *β*_*t *_is referred to as the score *S*_*j*_[12]. In this way, the score *S*_*j *_expresses the degree of importance for the *j*-th peak in terms of contribution to integrating a final classification function in the process of learning algorithm.

**Figure 5:**
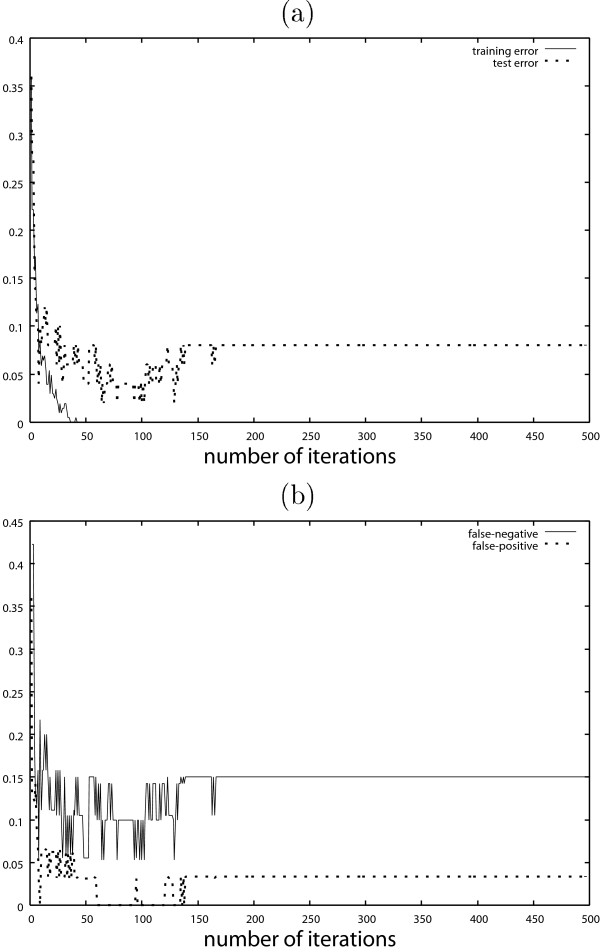
Results obtained by AdaBoost with continuous covariates. (a) Training error and test error (b) False negative and false positive.

**Figure 6:**
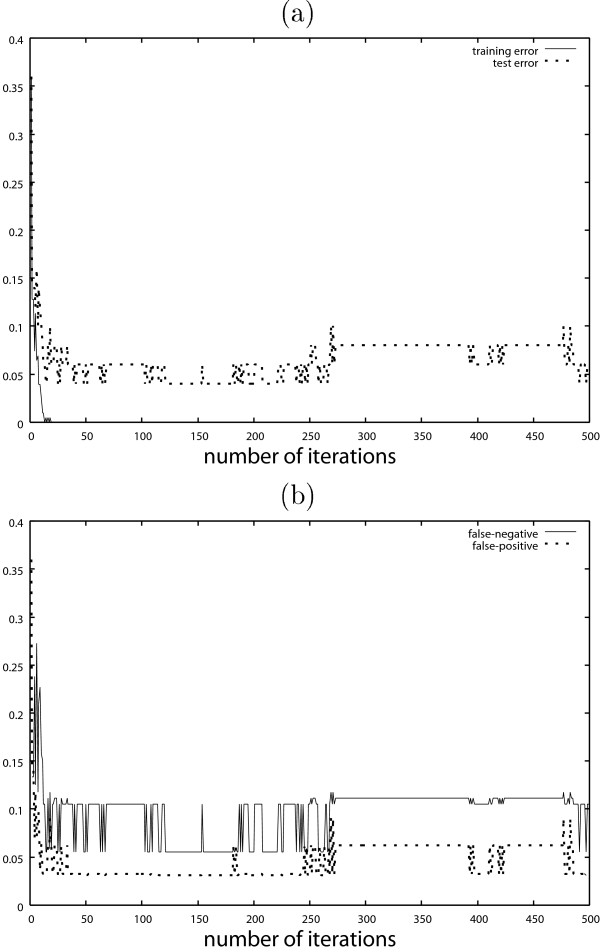
Results obtained by AdaBoost with continuous covariates. (a) Training error and test error (b) False negative and false positive.

Figure 5 (a) shows the test error of the classification result by AdaBoost with the discrete covariates. The test error is calculated by the test dataset with *n *= 50 observations separated from the training dataset. Figure 5 (b) shows the false negative and false positive results. The results obtained by AdaBoost with the continuous covariates are shown in Fig. 6. These figures show typical behaviors of AdaBoost. In Figs. 5 (b) and 6 (b), the false negatives are much larger than the false positives. Table 1 shows the test error of AdaBoost, where the iteration number *T *is decided by 10-fold cross validation.

In Table 1, the test error with discrete covariates and that with continuous covariates are the same.

The score *S*_*j *_is calculated to consider the difference between the obtained prediction functions. Each *S*_*j *_gives the influence of the *j*-th covariate for the obtained classification function. Tables 2 and 3 show the ten highest values among *S*_*j*_'s in the discrete and continuous cases, respectively. From Tables 2 and 3, the prediction functions obtained using the discrete and continuous variables are different. The result obtained using continuous covariates appears to be based on a difference in the intensity under the condition that the peak exists, but the result obtained using discrete covariates is likely to be based on a rougher difference whether the peak exists at the *m*/*z-*value. In this dataset, there may be many peaks that affect classification, but each individual peak will not have sufficient information to perfectly distinguish cancer patients from controls. In such a situation, the continuous covariates with high score and the discrete covariates with high score are different, but a classification function with high predictive performance will be obtained by combining information from many peaks by either continuous covariates or discrete covariates.

The above results were obtained with *h*_th _= 0.1. We can use more (or fewer) covariates by setting a smaller (or larger) *h*_th_. Tables 4 and 5 show the results for various values of *h*_th _in the discrete and continuous cases, respectively. When *h*_th _is large, the test error becomes larger in the discrete case, whereas in the continuous case, the test error does not vary as much.

In Figs. 5 and 6, the false negatives are much larger than the false positives, but this is not a desirable result. In order to suppress false negatives, AsymBoost [13] may be useful. In AdaBoost, the loss function is given by (2), but in AsymBoost, the loss function is given by

∑i=1Nwiexp⁡[−yi{β1f1(xi)+⋯+βTfT(xi)}],     (3)
 MathType@MTEF@5@5@+=feaafiart1ev1aaatCvAUfKttLearuWrP9MDH5MBPbIqV92AaeXatLxBI9gBaebbnrfifHhDYfgasaacH8akY=wiFfYdH8Gipec8Eeeu0xXdbba9frFj0=OqFfea0dXdd9vqai=hGuQ8kuc9pgc9s8qqaq=dirpe0xb9q8qiLsFr0=vr0=vr0dc8meaabaqaciaacaGaaeqabaqabeGadaaakeaadaaeWbqaaiabdEha3naaBaaaleaacqWGPbqAaeqaaaqaaiabdMgaPjabg2da9iabigdaXaqaaiabd6eaobqdcqGHris5aOGagiyzauMaeiiEaGNaeiiCaaNaei4waSLaeyOeI0IaemyEaK3aaSbaaSqaaiabdMgaPbqabaGccqGG7bWEiiGacqWFYoGydaWgaaWcbaGaeGymaedabeaakiabdAgaMnaaBaaaleaacqaIXaqmaeqaaOGaeiikaGIaemiEaG3aaSbaaSqaaiabdMgaPbqabaGccqGGPaqkcqGHRaWkcqWIVlctcqGHRaWkcqWFYoGydaWgaaWcbaGaemivaqfabeaakiabdAgaMnaaBaaaleaacqWGubavaeqaaOGaeiikaGIaemiEaG3aaSbaaSqaaiabdMgaPbqabaGccqGGPaqkcqGG9bqFcqGGDbqxcqGGSaalcaWLjaGaaCzcamaabmGabaGaeG4mamdacaGLOaGaayzkaaaaaa@60E3@

where each initial weight *w*_*i *_is set as follows:

wi={α,if the i-th patient is cancer,1,otherwise.
 MathType@MTEF@5@5@+=feaafiart1ev1aaatCvAUfKttLearuWrP9MDH5MBPbIqV92AaeXatLxBI9gBaebbnrfifHhDYfgasaacH8akY=wiFfYdH8Gipec8Eeeu0xXdbba9frFj0=OqFfea0dXdd9vqai=hGuQ8kuc9pgc9s8qqaq=dirpe0xb9q8qiLsFr0=vr0=vr0dc8meaabaqaciaacaGaaeqabaqabeGadaaakeaacqWG3bWDdaWgaaWcbaGaemyAaKgabeaakiabg2da9maaceqabaqbaeaabiGaaaqaaGGaciab=f7aHjabcYcaSaqaaiabbMgaPjabbAgaMjabbccaGiabbsha0jabbIgaOjabbwgaLjabbccaGiabdMgaPjabb2caTiabbsha0jabbIgaOjabbccaGiabbchaWjabbggaHjabbsha0jabbMgaPjabbwgaLjabb6gaUjabbsha0jabbccaGiabbMgaPjabbohaZjabbccaGiabbogaJjabbggaHjabb6gaUjabbogaJjabbwgaLjabbkhaYjabcYcaSaqaaiabigdaXiabcYcaSaqaaiabb+gaVjabbsha0jabbIgaOjabbwgaLjabbkhaYjabbEha3jabbMgaPjabbohaZjabbwgaLjabb6caUaaaaiaawUhaaaaa@687B@

**Figure 7:**
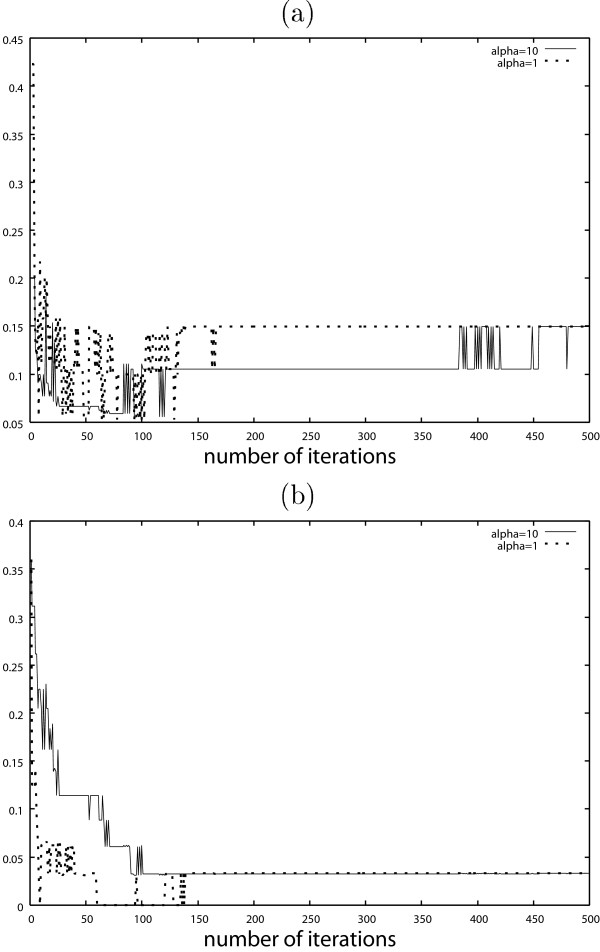
Results obtained by AsymBoost with discrete covariates when *α *= 1 and *α *= 10. (a) False negative, (b) False positive.

**Figure 8:**
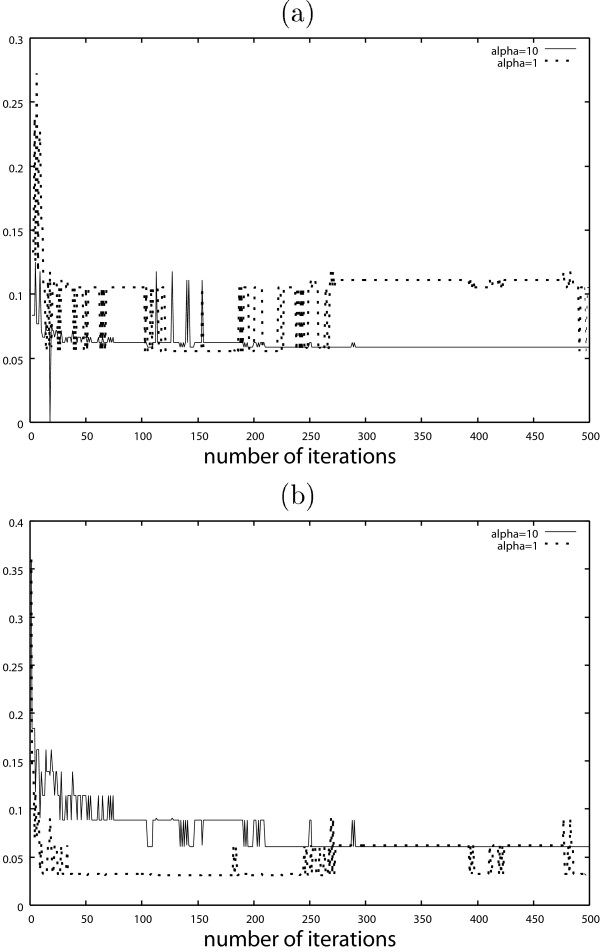
Results obtained by AsymBoost with continuous covariates when *α *= 1 and *α *= 10. (a) False negative, (b) False positive.

It is expected that the false negatives will be suppressed by using a large *α*. In Figs. 7 and 8, the false negatives might be suppressed by using a large *α *at the expense of the larger false positives, in particular when the number of iterations is small.

## Conclusion

We proposed a methodology for identifying biomarkers from high-dimensional mass spectrometry data. "Common" peaks in the data are regarded as biomarkers. The number of biomarkers can be changed by varying the value of *h*_th_, which is a threshold value that controls how "common" peaks can be regarded as biomarkers. By identifying biomarkers, the number of covariates is reduced, so that classification is facilitated. We can select discrete or continuous covariates depending on the situation.

The effectiveness of our approach was demonstrated through application to an ovarian cancer dataset. It was shown that a prediction function with high performance can be obtained by a simple application of AdaBoost.

A simple method was used to analyze data in this study. In general, however, a more sophisticated method may be required to extract a covariate at a biomarker. For example, when discrete covariates are extracted, we can use peaks obtained by a stricter rule or we can extract variables effective for classification [5]. When continuous variables are extracted, we can use the intensity at the *m*/*z*-value nearest to a biomarker, or the average of intensities within a window including a biomarker.

In this paper, the difference between the result for the continuous covariates and that for the discrete covariates was investigated in detail. In the example, the result obtained using continuous covariates appeared to be based on a difference in the intensity under the condition that the peak exists, but the result obtained using discrete covariates was likely to be based on a rougher difference whether the peak exists at the *m*/*z*-value. In general, whether discrete covariates are better or continuous covariates are better depends on data. If the value of the intensity in the data is reliable, it may be better to use continuous covariates. If not, it may be better to use discrete covariates. We consider that both cases of covariates should be examined and the results compared and inspected in detail for practical almost studies. We conclude that we can obtain more information on the structure of the data by integrating both results.

## Authors' contributions

TF wrote programs for this study and main parts of the manuscript. HF wrote a half of the section of Background. SE proposed the use of Asymboost when the false negative is small, and wrote the explanation on AdaBoost algorithm. All authors read and approved the final manuscript.

## Figures and Tables

**Table 1:** Test errors, false negatives and false positives.

	test error	false negative	false positive
discrete	0.06	0.11	0.03
continuous	0.06	0.11	0.03

**Table 2:** Ten highest values of *S*_*j *_in discrete case.

peak(*m*/*z*)	*S*_*j*_
3675	1.42
13651	1.06
7247	1.02
4907	1.00
7539	0.82
8569	0.76
4792	0.65
16668	0.62
1849	0.61
4728	0.54

**Table 3:** Ten highest values of *S*_*j *_in continuous case.

peak(*m*/*z*)	*S*_*j*_
17095	3.98
14798	2.46
7247	1.97
2095	1.68
4029	1.62
5271	1.56
8039	1.38
4118	1.19
4773	1.07
15044	1.05

**Table 4:** Test error when *h*_th _varies in discrete case.

*h*_th_	test error	number of variables
0.0	0.04	232
0.1	0.06	146
0.2	0.08	128
0.4	0.16	102
0.8	0.1	69

**Table 5:** Test error when *h*_th _varies in continuous case.

*h*_th_	test error	number of variables
0.0	0.06	232
0.1	0.06	146
0.2	0.06	128
0.4	0.08	102
0.8	0.12	69
